# Integrated transcriptome and proteome analyses identify novel regulatory network of nucleus pulposus cells in intervertebral disc degeneration

**DOI:** 10.1186/s12920-021-00889-z

**Published:** 2021-02-03

**Authors:** Chen Xu, Shengchang Luo, Leixin Wei, Huiqiao Wu, Wei Gu, Wenchao Zhou, Baifeng Sun, Bo Hu, Hongyu Zhou, Yang Liu, Huajiang Chen, Xiaojian Ye, Wen Yuan

**Affiliations:** 1 Spine Center, Department of Orthopaedics, Changzheng Hospital, Naval Medical University, Shanghai, 200003 China; 2 Microsurgery Center, Department of Orthopaedics, Changzheng Hospital, Naval Medical University, Shanghai, 200003 China; 3 Department of Orthopaedics, The First People’s Hospital of Huzhou, Huzhou, 200003 China; 4 Department of Orthopaedics, Tongren Hospital, Shanghai Jiao Tong University, Shanghai, 200050 China

**Keywords:** Intervertebral disc degeneration, Nucleus pulposus, Proteomics, Transcriptome, Bioinformatic analysis

## Abstract

**Background:**

Degeneration of intervertebral disc is a major cause of lower back pain and neck pain. Studies have tried to unveil the regulatory network using either transcriptomic or proteomic analysis. However, neither have fully elucidated the exact mechanism of degeneration process. Since post-transcriptional regulation may affect gene expression by modulating the translational process of mRNA to protein product, a combined transcriptomic and proteomic study may provide more insight into the key regulatory network of Intervertebral disc degeneration.

**Methods:**

In order to obtain the proteomic and transcriptomic data, we performed label-free proteome analysis on freshly isolated nucleus pulposus cells and obtained transcriptome profiling data from the Gene Expression Omnibus repository. To identify the key regulatory network of intervertebral disc degeneration in nucleus pulposus cells, we performed bioinformatic analyses and established a protein-RNA interacting network. To validate the candidate genes, we performed in vitro experimentation and immunochemistry labeling to identify their potential function during nucleus pulposus degeneration.

**Results:**

The label-free proteome analysis identified altogether 656 proteins, and 503 of which were differentially expressed between nucleus pulposus cells from degenerated or normal disc cells. Using the existing nucleus pulposus transcriptomic profiling data, we integrated the proteomic and transcriptomic data of nucleus pulposus cells, and established a protein-RNA interacting network to show the combined regulatory network of intervertebral disc degeneration. In the network, we found 9 genes showed significant changes, and 6 of which (CHI3L1, KRT19, COL6A2, DPT, TNFAIP6 and COL11A2) showed concordant changes in both protein and mRNA level. Further functional analysis showed these candidates can significantly affect the degeneration of the nucleus pulposus cell when altering their expression.

**Conclusions:**

This study is the first to use combined analysis of proteomic and transcriptomic profiling data to identify novel regulatory network of nucleus pulposus cells in intervertebral disc degeneration. Our established protein-RNA interacting network demonstrated novel regulatory mechanisms and key genes that may play vital roles in the pathogenesis of intervertebral disc degeneration.

## Background

Lower back pain and neck pain are common musculoskeletal disorders that could result in severe social and economic burdens on patients [1]. Current studies and epidemiologic investigations [2, 3] showed that these symptoms are closely related to intervertebral disc degeneration (IDD) mediated neurological defects. IDD is initiated by a series of pathogenic processes including biological, biochemical and structural impairment that eventually causes imbalanced metabolism of the extracellular matrix (ECM) of the intervertebral disc, thus manifesting as collapsed disc and incapability of maintaining movement. The nucleus pulposus (NP) region of the disc is most vulnerable during these pathogenic processes, and such degeneration affect its normal function of absorbing the pressure and maintaining the flexibility of the spine. The etiology is rather complex and multifactorial, in which aging, injuries, and genetic variations are all involved. Although many factors are associated with the IDD, the exact mechanism is far from clear. Hindered by the poor understanding of its molecular mechanism, no effective non-surgical treatment has been developed to treat IDD. Thus, a better understanding of the pathophysiology and mechanism of IDD will provide fundamental basis on developing novel non-surgical treatments to prevent or cure degenerated NP cells from patients with IDD, and to increase the quality of life.

Since the initial degeneration change appears in the nucleus pulposus (NP) region that compromises the function of imbibing water, leading to a decrease in the intradiscal pressure [4]. Subsequently, the swollen NP altered the transfer of gravity load and formed protrusion by the increasing static pressure to the rear of the disc, causing spinal cord or nerve root compression [5]. The annulus fibrosus (AF) functions differently from NP as their respective constituents or extracellular matrix (ECM) components are very different [6]. While NP is mainly composed of type II collagen and aggrecans to provide basis for forming the osmotic environment for optimal disc hydration, the AF is mostly composed of type I collagen optimized for resisting tensile load [6]. As their constituents alters, the degeneration of intervertebral disc occurs. During degeneration, the components like proteoglycans and type II collagen of the NP decreased significantly, while increased synthesis of collagen I was found, and resulted in decreased glycosaminoglycan/ hydroxyproline ratio [7].

Studies showed multiple factors could affect the degeneration process, such as increased activities and expressions of extracellular matrix metabolic enzymes such as matrix metalloproteinases (MMPs), TIMPs, inflammatory molecules (such as interleukin-1, interleukin-6, interleukin-8 and tumor necrosis factor α) [8, 9]. On the other hand, many anabolic factors like insulin-like growth factor (IGF-1) and its receptor (IGF-1R), chondroitin sulfate synthase 1 (CHSY-1), chondroitin sulfate N-acetylgalactosaminyltransferase 1 (CSGALNACT1) are found to be decreased significantly during IDD [10]. However, these factors alone are far from fully revealing the regulatory network of IDD. Recently, high through-put sequencing technologies have been used to identify critical regulatory factors in many biological processes through bioinformatic analysis. Many studies have used this technology to decipher the regulatory network of IDD [11–13]. Intriguingly, the results of which varies significantly between the studies. Such phenomenon was mainly thought to be caused by sample variation or other potential technical bias. However, recent studies purposed that post-transcriptional modifications were also important mechanism that take part in the pathogenesis of IDD, which add to the complexity of the regulatory network of IDD [14]. Thus, either transcriptomic or proteomic data alone would not be sufficient to elucidate the precise regulatory mechanism and uncover novel critical factors of IDD. A more comprehensive global analysis study is needed to solve this problem.

mRNA translation involves mechanism such as post-transcriptional or translational regulation. Disturbances of the expressions of these mechanism related factors may cause uneven translation of mRNAs, thus causing global expression change [15]. Taking this into account, we combined the proteomic profiling data with transcriptomic profiling data of NP cells from degenerated intervertebral discs to acquire a more comprehensive global regulatory network, and compared these the differences and key regulators of IDD.

## Methods

### Nucleus pulposus tissue collection and primary cell culture

Informed consent was obtained and signed by both the patient and their relatives before acquiring the intervertebral disc tissue during surgery as requested by the ethics committee in our institute. The experimental protocol was also authorized by the ethics committee of Naval Medical University. Magnetic resonance Imaging (MRI) T-2 weighted images were used to evaluate and diagnose IDD according to the modified Pfirrmann grading system [3]. Relatively normal intervertebral disc tissue samples (n = 3) were collected from lumbar spine trauma patients who underwent spinal fusion that need to remove relatively normal intervertebral disc (Pfirrmann grade I, n = 3). Patients were aged from 45 to 49 years old (mean 47 years). The degenerated intervertebral disc tissue samples were collected from lumbar disc herniation patients (n = 3) diagnosed by MRI, whom of which underwent disc resection and fusion surgery (Pfirrmann grade IV–V, n = 3). Patients were aged from 46 to 50 years old (mean 48 years).

For primary cell extraction, disc tissue specimens were washed twice with PBS, the NP region of the disc was isolated under a microscopy and then minced and digested with 2 U/mL protease in DMEM/F12 (Gibco, Grand island NY, USA) for 30 min at 37 °C [3]. The samples were further treated with 0.25 mg/mL type II collagenase (Gibco, USA) for 4 h at 37 °C [3]. Cell suspension was then transferred through a cell strainer (BD Falcon, NJ, USA) and centrifuged at 800 g for 5 min [3]. The cell pellets were used subsequently in proteomic analysis or total RNA extraction [3].

For gene knockdown, we used synthesized siRNAs (Shanghai GenePharma Co., Ltd, Shanghai, China) to perform siRNA mediated knockdown on the candidate genes [11]. The transfections of the siRNAs were performed using Silencer ™ siRNA Transfection II Kit (Thermofisher, CA, USA) according to the manufacturer’s protocol, and treated cells were harvested for further analysis after 48 h [11]. The sequences of the siRNAs can be found in Additional file 1.

### RNA reverse transcription

Trizol (Invitrogen, Carlsbad, CA) was used to extract the total RNA from human nucleus pulposus cell samples according to the manufacturer’s instructions as previously reported [11]. Total RNA extracts were measured at 260 nm with a spectrophotometer to identify the concentration (DU-800; Beckman Coulter, Brea, CA, USA). The reverse transcriptions were done by first generating the complementary DNA (cDNA) in a 10μL final volume containing 500 ng total RNA using Primer Script RT Master Mix (Takara, Japan) according to manufacturer’s instructions as previously reported [11].

### Quantitative polymerase chain reaction

Real-time polymerase chain reaction (RT-PCR) analysis of CHI3L1, KRT19, CLIP, COL6A2, DPT and COL11A2 were performed using SYBR premix Ex Taq™ (Takara Bio Inc., Shiga, Japan) in a Step One Plus real-time PCR system (Applied Biosystems, CA, USA) according to the manufacturer’s instructions as previously reported [11]. The amount of GAPDH analyzed was used to normalize the gene expressions to serve as internal control, and the relative number of transcripts was calculated using the comparative 2^–∆∆CT^ method [11]. Primers sequences were listed in Additional file 1. The mean values (± SD) were compared for statistical significance using a Student's t test for independent groups. *P* < 0.05 was assumed for significance in differences, and all experiments were repeated at least for three times independently.

### Label-free proteomic profiling

NP cell samples were centrifuged at 10,000×*g* for 30 min at 4 °C, and 100 μL lysis buffer (7 M urea, 2 M thiourea) were added into each sample followed by ultrasonication to extract the total proteins of NP cells [16]. Trichloroacetic acid (TCA) was used to precipitate proteins for 30 min at 4 °C and centrifuged at 40,000×*g* for 30 min. The concentration was measured according to the manufacturer’s instructions using Qubit quantification kit (Invitrogen, CA, USA) to determine the concentration [16].

Label-free proteomic profiling of the protein extracts were done by Beijing BangFei Bioscience Co., Ltd. In brief, 60 μg protein samples were used and addition of 100 mmol/L DTT (DL-Dithiothreitol) to its final concentration of 10 mmol/L was performed and mixed at 37 ℃ for 60 min. The mix was then diluted with 250 mmol/L IAM (Iodoacetamide) and kept in dark for 60 min [17]. 100 μL UA buffer (8 M urea, 100 mM Tris–HCl, pH8.0) were used to wash the samples for two times, and followed by three washes with 50 mM NH4HCO3 100 μl. Lastly, samples were digested with trypsin for 12 h at 37 ℃, and the supernatant were loaded onto a silica capillary column packed with 3-μm dionex C18 material [17]. Agilent 1100 quaternary HPLC (High Performance Liquid Chromatography) was used to analyze the sample using a 12-step separation after desalting [17]. Mass spectrometer was then operated in the MS/MS (auto) mode to acquire the raw data. The data were further processed and expressed proteins with *p* < 0.05 and signal more > 1.5-fold were considered as differentially expressed proteins.

### Transcriptome data acquiring and bioinformatics analysis

We downloaded the processed data of GSE70362 [13] from Gene Expression Omnibus (GEO) database for further transcriptome data analysis under the help of Shanghai NovelBio Bio-Pharm Technology Co., Ltd. The dataset uses NP cells from donor disc tissues for RNA profiling analysis [13]. Samples were rearranged according to Pfirrmann grade, which Pfirrmann grade I and II samples were classified as normal NP cells named IVD group, and Pfirrmann grade III-V were classified as degenerated NP cells named IDD group. The processed log_2_-transformed and quantile-normalized signal data were used, and genes with *p* < 0.05, signal fold-change ≥  ± 1.5 were filtered and considered as differentially expressed genes. Unsupervised hierarchical clustering was performed to detect the expression pattern change using the profiling data by average linkage and median centering, the processed data was visualized with TreeView [11]. To construct the protein-mRNA regulatory network of IDD, we made use of normal NP cell with degenerated NP cell profiling data according to the gene’s mRNA and protein expression change (fold change) between the groups. Both the mRNA and the protein profiling data were used. The KCore method was used to calculated the relationship between the genes, and the correlation of the genes were calculated and presented using the Pearson correlation method. FDR and *P* value were used to selected candidate genes to form the network, and the presented network is further drawn according to the KCore value and the calculated Degree value. Other analysis like Gene Ontology (GO) analysis was carried out using annotations in PANTHER database v 6.1 (www.pantherdb.org) [11], and pathway analysis were performed with the tools on the KEGG database (http://www.genome.jp/kegg/pathway.html) respectively [11].

### Immunohistochemistry analysis

To localize the expression of DPT, COL6A2, KRT19, CLIP, COL11A2 and CHI3L1 in NP tissue samples, we made use of three normal (Pfirrmann grade 1, IVD group) and three degenerated NP tissue samples (grade IV-V, IDD group) for the immunohistochemistry analysis as previously reported [11]. The sections were incubated with rabbit polyclonal antibody against DPT, COL6A2, KRT19, CLIP, COL11A2 and CHI3L1 (1:200 dilution, all from Abcam). The goat anti-rabbit peroxidase-conjugated IgG (1:1000 dilution, Proteintech) was as secondary antibody, and the sections were counterstained with hematoxylin. The imaging of the sections uses a ZEISS microscopy (ZEISS Axio Imager A2, Carl Zeiss microscopy GmbH, Germany).

## Results

### Global proteome and transcriptome analysis of IDD

**Fig.1:**
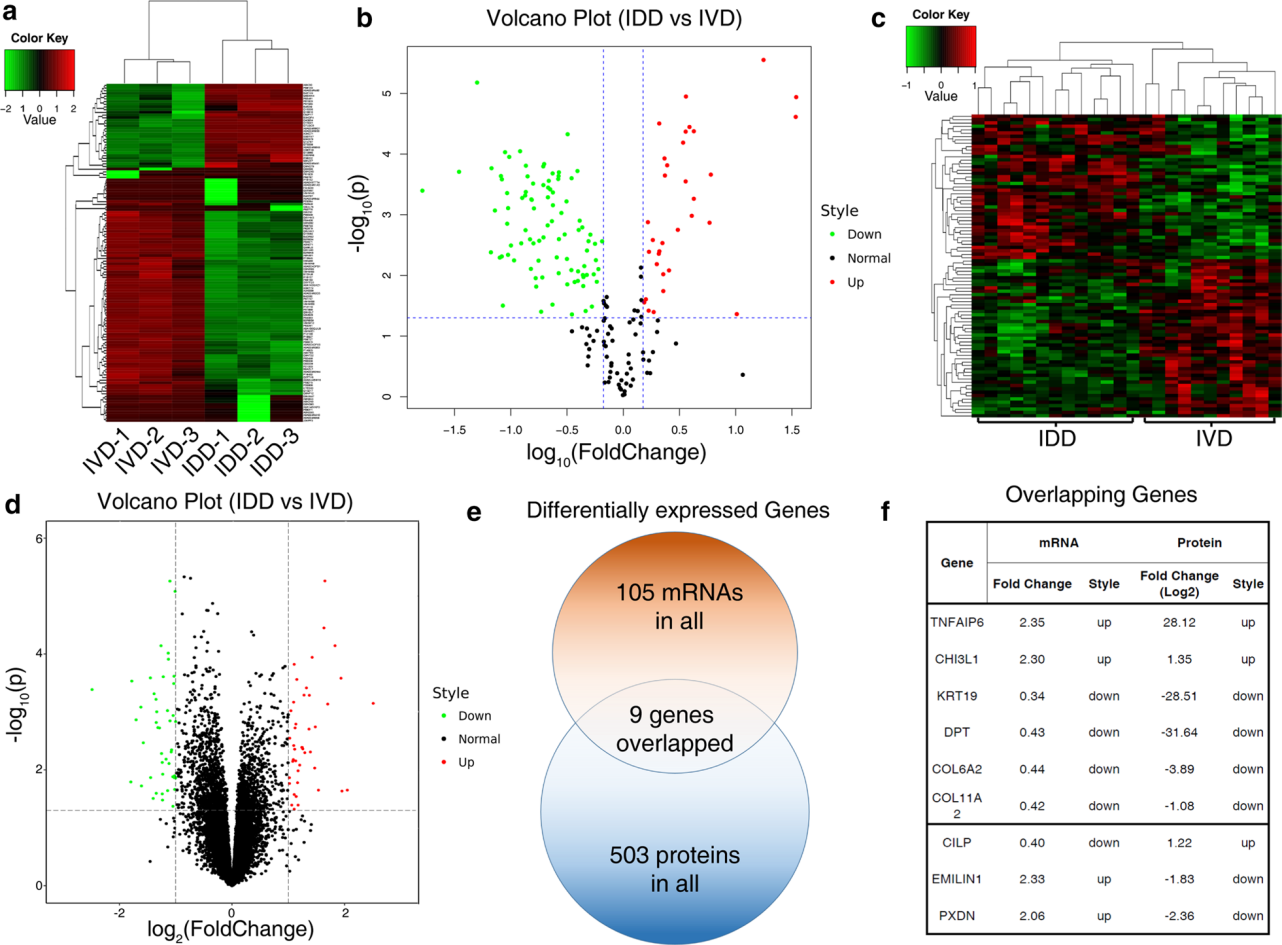
Assessing the differentially expressed mRNAs and proteins of NP cells in intervertebral disc degeneration. The unsupervised Hierarchical cluster plot of the label-free proteomic sequencing data (**a**) or RNA sequencing data (**c**) showing the differentially expressed proteins (**a**) or mRNAs (**c**) of NP cells from IVD and IDD groups. The green color indicates relatively lower expression of each gene, while the red color indicates higher expression. The volcano plot showing the degree of fold changes of each gene’s protein (**b**) or mRNA (**d**) expression in NP cells between IVD and IDD groups. **e** A Venn plot showing the number of genes that have significant changes in either protein level, mRNA level or both. **f** The list of the overlapped genes that are significantly changed during disc degeneration

To gain initial knowledge of the global protein and RNA expression pattern of primary NP cells during intervertebral disc degeneration (IDD), we first performed label-free proteomic analysis of IDD and normal NP cells. Three normal NP (Pfirrmann grade I, n = 3, age 45 to 49 years, mean age 47 years) and three IDD NP (Pfirrmann grade IV–V, n = 3, age 46 to 50 years, mean age 48 years) tissue samples were collected from male patients of similar ages, The NP cells were initially isolated to exclude further influences of protein degradation, and cells were extracted for proteomic profiling. In the data, we identified 656 proteins, and 503 of which were identified as differentially expressed proteins (> 2 Fold change in expression with *P* value < 0.05, Additional file 2, Fig. 1a, b), Here we compared the proteome data with transcriptome data using the existing GEO dataset GSE70362. We reanalyzed the transcriptome data and found 105 differentially expressed mRNA between degenerated and normal NP cells (Additional file 3). The Hierarchical clustering and volcano plot showed less variation between genes in the transcriptome data than that of proteome data (Fig. 1c, d). To show the concordance of both data, we compared the differentially expressed genes and found only 9 genes were both significant in protein and mRNA data (Fig. 1e), while only TNFAIP6, CHI3L1, KRT19, DPT, COL6A2 and COL11A2 showed concordant expression changes in both data (Fig. 1f, Additional file 4). These results implied that global mRNA changes were significantly different from global protein changes during IDD, and one gene’s mRNA level change may not reflect on the expression of its final protein product, which needs to pay more attention to when studying genes that affect IDD process.

### Functional analysis of differentially expressed genes

**Fig. 2:**
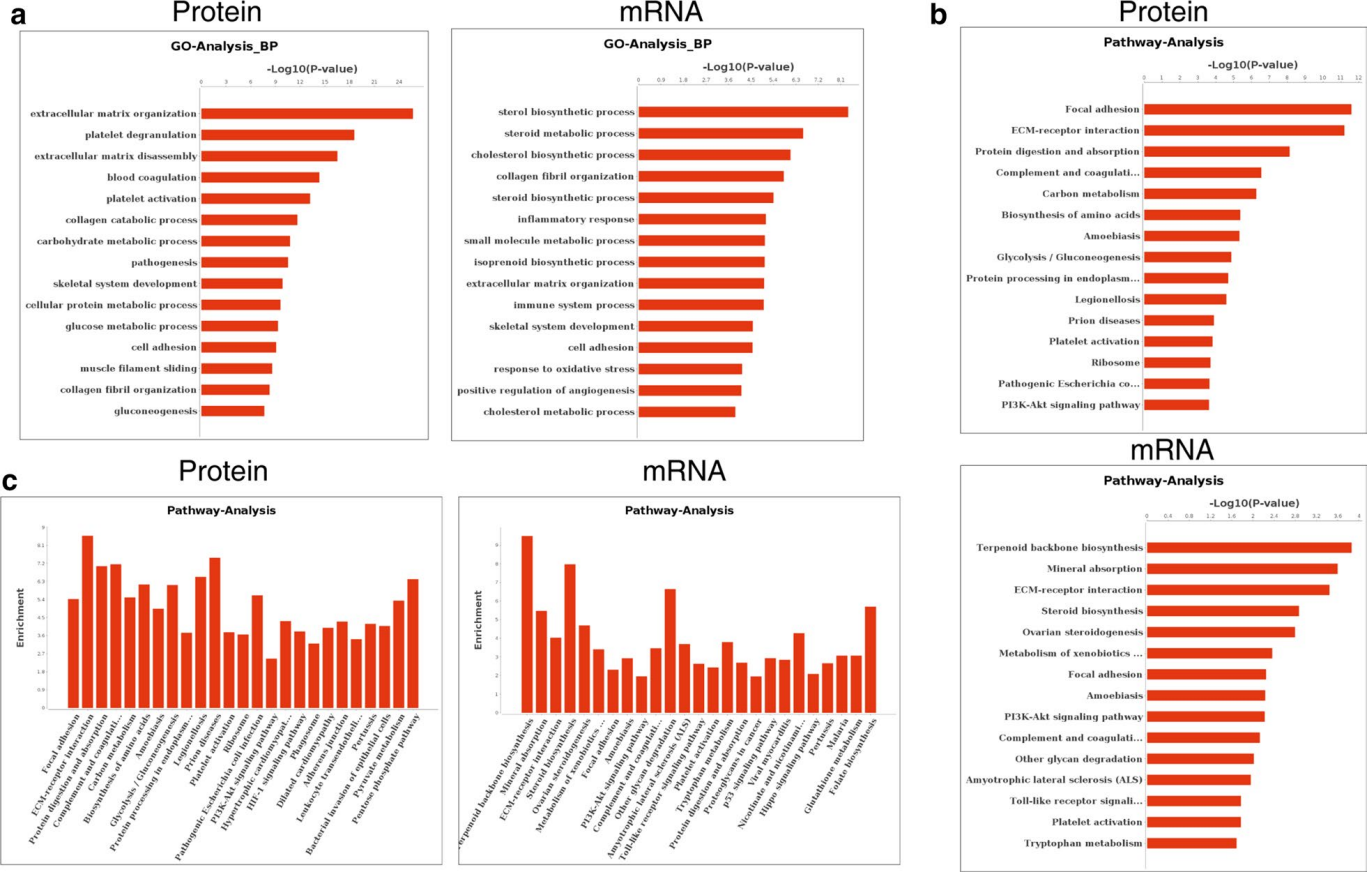
Functional analysis of differentially expressed proteins and mRNAs in the NP cells during intervertebral disc degeneration. **a** Gene Ontology (GO) analysis showing the significance (*P*-value) of each GO function categories according to the differentially expressed genes. Pathway analysis showing the **b** significance (*P*-value) or **c** enrichment of different pathways according to the differentially expressed genes

In order to identify the functions of these differentially expressed genes, we performed GO and Pathway analysis on both proteomic and transcriptomic data. By comparing the results we found that protein changes mainly focused on extracellular matrix related metabolism (GO:0030198, GO:0022617, GO:0030574, GO:0005975, GO:0,044,267, GO:0006006), while mRNA changes mainly focused on steroid and cholesterol metabolism (GO:0016126, GO:0008202, GO:0006695), extracellular matrix related metabolism (GO:0030198, GO:0030574, GO:0007155), stress and inflammatory response (GO:0006954, GO:0002376, GO:0006979) in Fig. 2a. In Pathway analysis (Fig. 2b, c), both data showed significant enrichment in ECM interaction, Focal adhesion and PI3K-Akt signaling pathways. However, only protein data showed significant enrichment in TGF-beta signaling pathway and HIF-1 signaling pathway (Additional file 5), which is concordant with previous reports [2, 18, 19]. These findings showed that the changes of either transcriptome and proteome of the degenerated NP cells were indeed related to the extracellular matrix remodeling or degeneration, but there exist great differences in the enriched gene functions and pathways of the differentially expressed genes between mRNA and protein data. Such differences indicated that the gene interacting network for either transcriptome or proteome of IDD may be different, and a combined analysis is needed to uncover the underlying relationship between the proteome and transcriptome regulatory network of IDD.

### Co-expression network reveals vital regulatory genes in IDD

**Fig.3:**
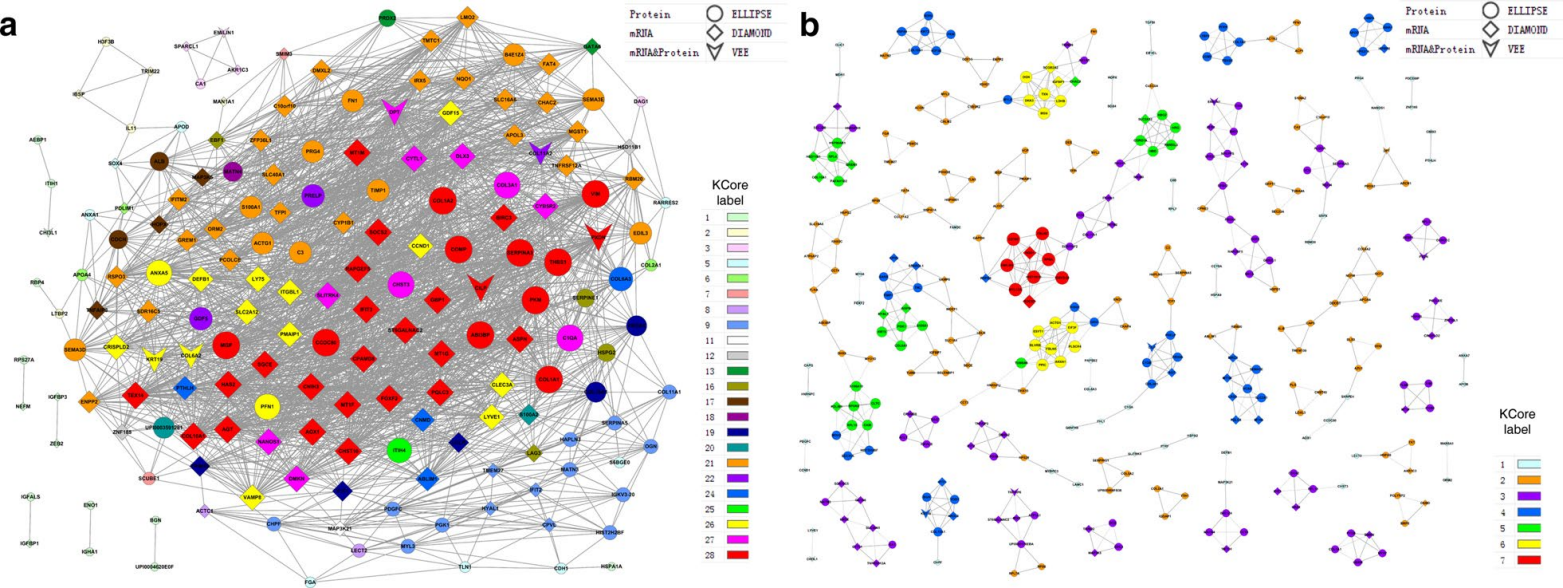
Protein and mRNA Co-expression network of normal and degenerated NP cells. The gene co-expression network (GCN) of IDD (**a**) or IVD (**b**) group are constructed using the differentially expressed mRNAs and proteins according to their expression level. Different shapes of the nodes represent a gene that is either differentially expressed in protein, mRNA or both protein and mRNA level in the IDD group. The index of K-Core represents different proposed interaction groups, the importance of each color group raises with K-Core values

Here, we construct the co-expression network to clarify the key component and potential relationship between these differentially expressed mRNAs and/or proteins. By comparing the normal and degenerated NP data, we constructed a co-expression network according to the mRNA or protein expression trend (fold change). From the network we can found that the degenerated co-expression network is rather focused, with each key node (gene) inter-connected with another, forming one big network loop (Fig. 3a). However, the co-expression network for normal NP is rather scattered compared to the degenerated NP network (Fig. 3b). Each node (refers to one differentially expressed gene in normal NP cells) was connected to few other nodes, forming a rather scattered and non-connected network in normal NP cells. This loosened network of normal NP cells indicated that these key genes may function by themselves and do not influence the others in maintaining the normal function of NP cells, while the degenerated NP cell network showing a more interconnected relationship that each differentially expressed gene will influence the expression of others during degeneration process. This phenomenon indicates that each gene in the IDD network may well play important roles during disc degeneration.

From the degenerated NP co-expression network, we found only 9 genes showed significant correlation with other genes both in mRNA and protein forms (CILP, PXDN, COL11A2, KRT19, DPT, EMILIN1, COL6A2, CHI3L1 and TNFAIP6), and only 6 of them (CHI3L1, KRT19, DPT, COL6A2, TNFAIP6 and COL11A2) showed concordant changes between mRNA and protein level during degeneration. This finding indicated the important functional roles of these candidate genes during IDD. Among which, KRT19 and COL6A2 were considered as important degeneration markers in the previous studies [20, 21], while the SNPs and mutations of COL11A2 are also linked to disc degeneration [22]. On the other hand, the functional roles of TNFAIP6, DPT, PXDN, CLIP, CHI3L1 and EMILIN1 in IVD are not elucidated yet. Since the network is constructed by KCore and Degree value (Additional file 6), it is obvious that centered nodes are more intercorrelated with other genes, which may take an important functional role in the degeneration phenotype. Among which, CHST3, COL1A2, TIMP1, COMP, CCDC80 and ABI3BP are most centered proteins in the degenerated network. CHST3, TIMP1 and COL1A2 are known genes correlated with IDD [23–25], while the correlation of COMP, CCDC80 and ABI3BP with IDD are not elucidated yet. Aside from proteins, ASPN, ITGBL1 and IFIT3 are of the few key mRNAs that are correlated with IDD in previous reports [26–29]. While many other key mRNAs are not reported to be related with IDD. By further analyzing the network, we can find ossification related genes, IBSP, SOX4, RSPO3 and OGN are also included in the network. The possible functions of these ossification related genes have not been studied yet except for RUNX2 reported in mice IDD [30], which implies that they may also play a vital role in IDD.

### Verification analysis of candidate genes in IDD tissue sample

**Fig. 4:**
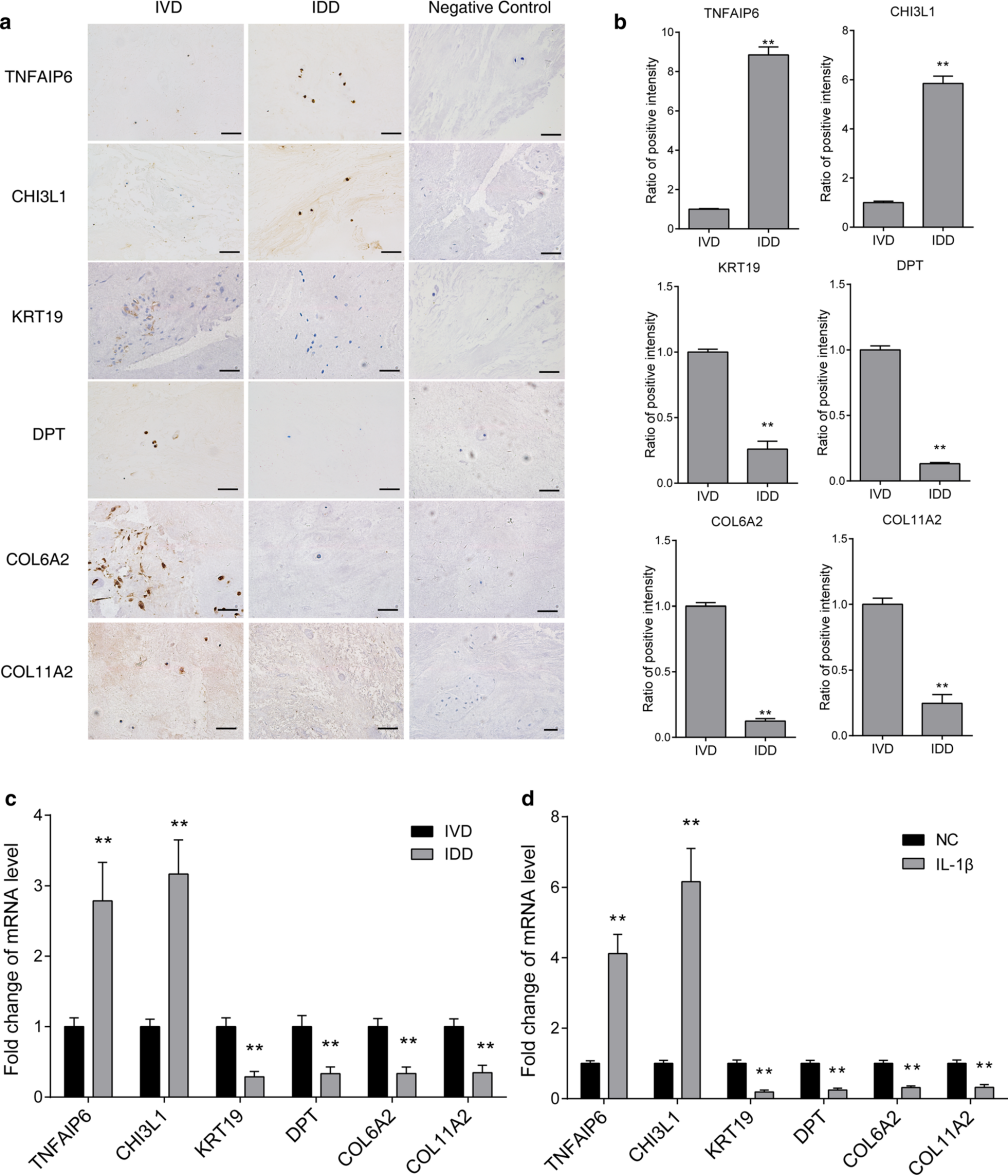
Verification of the expression of candidate genes in normal (IVD) and degenerated (IDD) NP primary cells. **a** The expression of TNFAIP6, CHI3L1, KRT19, DPT, COL6A2, and COL11A2 were examined in normal (IVD, n = 6) and degenerated (IDD, n = 6) NP tissues using immunohistochemistry. **b** The quantifications of the immunohistochemistry analysis were performed to show the relative expression level of the candidate genes. **c** The RNA expression level of these candidate genes in normal (IVD, n = 3) and degenerated (IDD, n = 3) NP primary cells were assessed using real-time PCR method. **d** The RNA expression level of these candidate genes in normal (NC, n = 3) and IL-1β (50 ng/ml, resembles the degeneration process in vitro, n = 3) treated NP primary cells were assessed using real-time PCR method. RNA level of GAPDH was used as internal reference. All Data are represented as mean ± SD, **p < 0.01

To further validate the interacting network, we first examined the expression of a few candidates. Here, we selected TNFAIP6, CHI3L1, KRT19, DPT, COL6A2 and COL11A2 as candidate genes for further validation for they showed concordant changes in both RNA and protein level in NP degeneration, which may potentially exert significant function in IDD process. By immunohistochemistry, we found that the expression levels of the candidates were all consistent with the transcriptomic and proteomic sequencing data (Fig. 4a, b). Using real-time PCR analysis, we also confirmed the expression changes of the candidate genes between normal and degenerated NP samples derived primary NP cells were consistent with the sequencing results (Fig. 4c). Using the in vitro NP cell degeneration model by adding IL-1β to normal NP cells, we found that the expression changes of these candidates became more drastic than previously found (Fig. 4d). Taken together, these expression validations showed consistent findings with the sequencing data, and implied vital roles of these candidate genes in the process of degeneration.

**Fig. 5:**
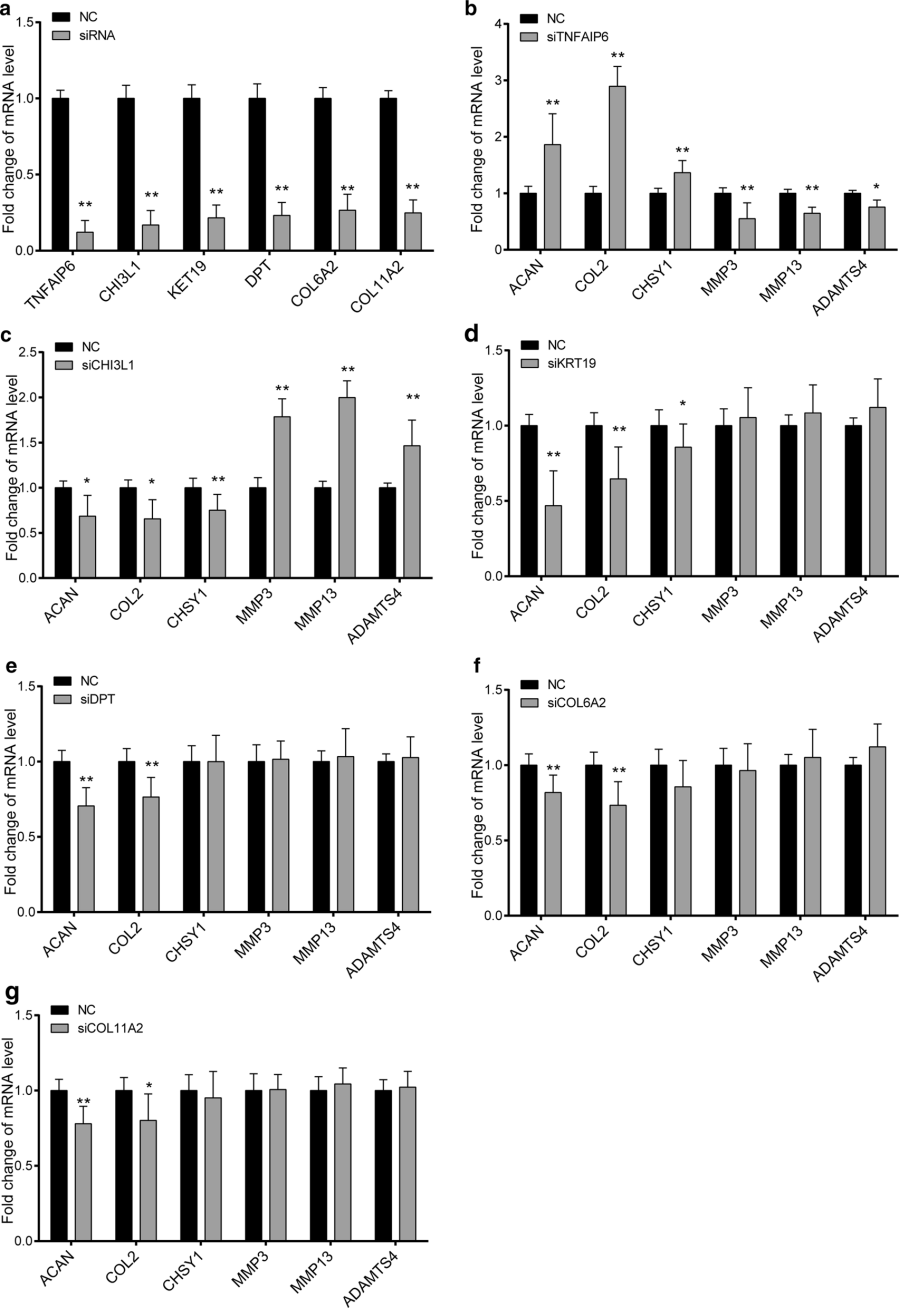
Functional study of candidate genes in degenerated NP primary cells. **a** The efficiency of TNFAIP6, CHI3L1, KRT19, DPT, COL6A2, and COL11A2 knockdown were examined in normal NP cells. NC group represents NP cell transfection using a scramble siRNA that showed no potential target to these genes. The RNA expression level of NP ECM related anabolic genes (ACAN, COL2 and CHSY1) and catabolic genes (MMP3, MMP13, ADAMTS4) were examined in TNFAIP6 (**b**), CHI3L1 (**c**), KRT19 (**d**), DPT (**e**), COL6A2 (**f**), and COL11A2 (**g**) knockdown NP cells using real-time PCR method. RNA level of GAPDH was used as internal reference. All Data are represented as mean ± SD, *p < 0.05, *p < 0.01

To further show their function in IDD, we designed small interfering RNAs targeting these candidates to perform knockdown in NP cells. The efficiency of the knockdown siRNAs was tested prior to functional examination (Fig. 5a). By using real-time PCR analysis, we found that TNFAIP6 and CHI3L1 knockdown showed significant changes in both NP extracellular matrix related anabolic and catabolic genes, while KRT19, DPT, COL6A2 and COL11A2 knockdown showed significant changes only in anabolic genes in IL-1β treated NP cells (Fig. 5b–g). These data indicated that KRT19, DPT, COL6A2 and COL11A2 may play roles in maintaining NP cell ECM production, while TNFAIP6 and CHI3L1 may influence both the normal ECM regulation and inflammation responses.

## Discussion

The degeneration of intervertebral disc is a complex biological process that the mechanism of which is less known [2]. Many researchers have been dedicated to uncover the specific mechanism, but aside from the well-known inflammation and cell apoptosis causes, the disease-specific trigger or disease-specific regulatory network that initiates the degeneration process is still not fully unveiled [2]. Many studies recently have taken use of high through-put profiling technologies to identify either transcriptomic or proteomic pattern changes of IDD [19, 31, 32], but few have untangled the critical regulatory mechanism between the differentially expressed proteins and mRNAs during the IDD process.

Recently, post-transcriptional regulation mechanism has been identified as important molecular mechanism that affect the pathological events in intervertebral disc degeneration process [3]. Post-transcriptional regulation refers to the regulation of efficiency of mRNA translation to protein products by various factors [11]. For example, microRNAs (miRNAs) are well-known post-transcriptional regulators of mRNA translation, and functions in many biological events. Conditional knockout of miRNA processing enzyme Dicer1 would cause translational defects of mRNAs and causes critical effect on cellular or mammalian survival [11]. Another typical model is natural antisense transcript, which is generated from the opposite DNA strand to other transcriptional genes [33]. These antisense transcripts are usually non-coding transcript that related to the antisense strand of a protein-coding gene [34], and could regulate that protein-coding gene expression at both transcriptional or post-transcriptional level through simple mRNA pairing of antisense transcripts to their sense strands to induce degradation [35]. Alternatively, antisense transcripts may cause blocking effect on translational process of the sense-strand mRNAs without causing degradation of the latter [36]. Thus, a transcriptomic and proteomic combined approach is needed to decipher the IDD mechanism in case of biased results that generated by either transcriptomic or proteomic approach along.

In order to testify this hypothesis, we those high through-put profiling data to gain insight into the regulatory network of intervertebral disc degeneration. In our constructed network, we found many unreported genes that had the potential to act as important regulators during the IDD process. Looking into the degenerated network we constructed, we can find the network is more condensed and interconnected with genes of many different functions, which indicated that the degeneration process may act in cascades. This means that all the included genes could affect each other and take a role in the pathogenesis of IDD in an uncovered mechanism. During which, we can find genes such as COL6A2, COL11A2, KRT19, COL1A2, TIMP1, and ITGBL1 are known extracellular matrix related factors in the regulation of IDD [22, 37, 38]. Since the most eminent change of disc degeneration takes part in the extracellular matrix region of nucleus pulposus, these genes could act as ‘effectors’ or downstream components resulted from the activation of NP degeneration cascades during degeneration [3]. Consequently, the upstream regulators of these ‘effectors’ could be more important to the process of degeneration [3]. And to reveal such regulators, we need to fully uncover the regulatory network of NP cells rather than searching the tissue for answers, which is what we did in this study using normal and degenerated NP cells for proteome and transcriptome study.

Among the network, we found enzymes that related to chondroitin sulfate (CS) biosynthesis and catalysis (CHST3, CHST10 and CHPF) showed significant changes during NP degeneration, which further support our previous findings [2, 3, 10, 14] in deciphering the vital role of CS regulation. However, these enzymes did not show concordant changes in both mRNA and protein expression, which is similar to our previous findings with CHSY [3]. Such phenomenon implied that post-transcriptional regulation (miRNA mediated or other mechanism) may play vital roles during the CS synthesis regulation as previously reported [3], further adds to the credibility of our results. Of the 9 genes that showed significant expression changes in both protein and mRNA data, only 6 of them showed concordant changes between the RNA and protein level. Similar to the case of CS regulation, the 3 genes that showed contradictory changes may also have the possibility of receiving post-transcriptional regulation which could be identified by further analysis. Thus, further assessment of the regulatory mechanism of these genes may help to understand the regulation mechanism of IDD.

Besides, we observed that osteogenesis related genes like IBSP, RSPO3, SOX4 and OGN are also potential regulators that were included in the degeneration related network, which indicated a vital role of ossification or osteogenesis related genes during the IDD pathogenesis. It has been reported that Runx2, a master transcriptional factor of chondrocyte hypertrophy and ossification, is the abundantly expressed in the nucleus pulposus cells during its degeneration [30]. The research also showed that Runx2 knock-in mice developed phenotypes of both ectopic mineralization and nucleus pulposus degeneration after birth, and strongly affect the normal function of intervertebral disc [30]. These results implied that ossification or osteogenesis related genes may well play as important regulators during the pathogenesis of intervertebral disc degeneration [30]. Thus, ossification or osteogenesis related IBSP, RSPO3, SOX4 and OGN we found could also act as potential key regulators that contribute to the generation of IDD, however, the specific function and detailed mechanism is not reported yet.

However, our study had limitations. First of all, the samples were collected from surgical patients, which would inevitably cause damage to the nucleus pulposus tissue, which would have effects on the gene expression of the NP cells (especially using trauma samples). So, the tissue samples, especially the normal NP samples, would have some differences to its physiological state. Moreover, the sample size for proteome study is rather small (only 3 control and 3 degenerated samples were used), which needs further validation for each candidate gene we reported in this study. Secondly, our study mainly focused on deciphering the interaction network of coding genes using RNA sequencing and protein sequencing data from NP cells, and due to the limitation of the techniques we used, some type of the proteins (like proteoglycans) and other functional RNAs like microRNAs and lncRNA (long non-coding RNAs) were not or could not be considered in this study. However, since the coding genes were of strong function in disease pathogenesis, we think that our data would throw light on further in depth studies based on our findings. Thirdly, due to the nature of the NP tissue, it is hard to collect large number of NP cells using one patient’s sample [39], so the limited cell number may hinder the discovery of low abundance proteins expressed in it. And also, due to the limited understanding of NP cell degeneration, other issues like selecting normalization genes would be difficult than normal. In the validation study, we examined only the candidate genes with concordant changes in both protein and RNA level during IDD, this does not mean other genes in the IDD network are not vital to the IDD process. Instead, others may well be valuable as these candidates in this process which need further verifications. Nevertheless, we provided novel regulatory network using combined proteomic and transcriptomic data, which may be of importance for further in depth study to decipher the actual network of IDD regulation.

## Conclusion

Taken together, our study provides new evidence and insights into the regulatory network of IDD. By combining the mRNA and protein profiling data, we purpose new IDD related interacting RNA–protein network and validated key regulators (TNFAIP6, CHI3L1, KRT19, DPT, COL6A2 and COL11A2) with potential functional roles in the IDD process, but the detailed mechanism is up to further investigation and validation.

## Supplementary Information

**Additional file: 1.** Oligonucleotide Sequences used in this study. The sequence of oligonucleotides used in this study.

**Additional file: 2.** Differentially expressed proteins in IDD. Contains the processed data of label-free proteomic profiling of normal (IVD) and degenerated (IDD) nucleus pulposus cells.

**Additional file 3.** Differentially expressed mRNAs in IDD. Contains the processed data of transcriptome profiling of normal (IVD) and degenerated (IDD) nucleus pulposus cells.

**Additional file 4.** Comparison of differentially expressed mRNA and Protein expressions. Annotation and comparison of processed protein and mRNA expression of normal and degenerated nucleus pulposus cells.

**Additional file 5.** GO and pathway analysis of differentially expressed protein and mRNA in IDD. Contains four sheet detailing the pathway and gene ontology analysis results of differentially expressed Protein and mRNA in IDD.

**Additional file: 6.** Degree value for network construction. The calculated value of gene degree for construction of protein-mRNA interacting network.

## Data Availability

The label-free proteomic profiling data can be found in the supplementary files. Additional supporting information on network construction and bioinformatic analysis can be found online in the supplementary files. The RNA expression datasets (Accession No. GSE70362) can be downloaded from the NCBI GEO database (https://www.ncbi.nlm.nih.gov/geo/).

## Abbreviations

IVD: Intervertebral disc
IDD: Intervertebral disc degeneration
mRNA: Messenger RNA
RNA: Ribonucleic acid
DNA: Deoxyribonucleic acid
GO: Gene ontology
cDNA: Complementary DNA
NP: Nucleus pulposus
AF: Annulus fibrosus
ECM: Extracellular matrix
GEO: The Gene Expression Omnibus
MRI: Magnetic resonance imaging
